# Diagnostic benefits of adding EspC, EspF and Rv2348-B to the QuantiFERON Gold In-tube antigen combination

**DOI:** 10.1038/s41598-020-70204-w

**Published:** 2020-08-06

**Authors:** R. Villar-Hernández, T. Blauenfeldt, E. García-García, B. Muriel-Moreno, M. L. De Souza-Galvão, J. P. Millet, F. Sabriá, A. Sánchez-Montalvá, J. Ruiz-Manzano, J. Pilarte, M. A. Jiménez, C. Centeno, C. Martos, I. Molina-Pinargote, Y. D. González-Díaz, J. Santiago, A. Cantos, I. Casas, R. M. Guerola, C. Prat, P. Andersen, I. Latorre, M. Ruhwald, J. Domínguez

**Affiliations:** 1 Servei de Microbiologia, Hospital Universitari Germans Trias I Pujol, Institut D’Investigació Germans Trias I Pujol, Badalona, Barcelona Spain; 2 CIBER Enfermedades Respiratorias, CIBERES, Instituto de Salud Carlos III, Madrid, Spain; 3 Universitat Autònoma de Barcelona, Barcelona, Spain; 4 Department of Infectious Disease Immunology, Centre for Vaccine Research, Statens Serum Institut, København S, Denmark; 5 Unitat de Tuberculosi de Drassanes, Hospital Universitari Vall D’Hebron, Barcelona, Spain; 6 Serveis Clínics, Unitat Clínica de Tractament Directament Observat de La Tuberculosi, Barcelona, Spain; 7 CIBER de Epidemiología Y Salud Pública, CIBEREESP, Instituto de Salud Carlos III, Madrid, Spain; 8 Servei de Pneumologia, Hospital Sant Joan Despí Moises Broggi, Sant Joan Despi, Barcelona Spain; 9 Infectious Diseases Department, Vall d’Hebron University Hospital, PROSICS Barcelona, Universitat Autònoma de Barcelona, Barcelona, Spain; 10 Grupo de Estudio de micobacterias (GEIM), Sociedad Española de Enfermedades Infecciosas y Microbiología Clínica (SEIMC), Barcelona, Spain; 11 Servei de Pneumologia, Hospital Universitari Germans Trias I Pujol, Barcelona, Spain; 12 Servei de Medicina Preventiva, Hospital Universitari Germans Trias I Pujol, Institut D’Investigació Germans Trias I Pujol, Barcelona, Spain; 13 Julius Center for Health Sciences and Primary Care, University Medical Center Utrecht, Utrecht University, Utrecht, The Netherlands

**Keywords:** Diagnostic markers, Tuberculosis

## Abstract

Interferon (IFN)-γ release assays (IGRAs) are used to diagnose latent tuberculosis (TB) infection (LTBI). To improve the accuracy of these tests, different approaches, such as alternative cytokine detection and using different antigens, are considered. Following this purpose, this study aims to evaluate the addition of EspC, EspF and Rv2348-B to those present in the QuantiFERON-TB Gold In-Tube (QFN-G-IT). We included 115 subjects: 74 active TB patients, 17 LTBI individuals and 24 healthy controls. Whole blood samples were collected in QFN-G-IT and in-house tubes containing different combinations of EspC, EspF and Rv2348-B, together with ESAT-6, CFP-10, and TB7.7. After overnight incubation at 37 ºC, plasma was harvested and IFN-γ quantified. IFN-γ levels in the QFN-G-IT and in-house tubes correlated very good (Spearman Rho(r) > 0.86). In-house antigen combinations distinguished healthy individuals from those with active TB and LTBI (specificities and sensitivities higher than 87.5% and 96.3%, respectively [AUC > 0.938]). Adding EspC, EspF and Rv2348-B, increased the sensitivity of the test, being the addition of EspC and Rv2348-B the combination that yielded a higher sensitivity with no specificity loss. Addition of these antigens could improve diagnosis in patients with impaired or immature immune response who are at high risk of developing TB.

## Introduction

Tuberculosis (TB) remains the leading cause of death by a single infectious agent^1^. Correct control, as well as proper management of the disease, are of great importance in order to decrease TB burden. For this, early diagnosis as well as correct and efficient preventive measures and anti-TB treatment, are key^2^. Screening of latent TB infection (LTBI) and preventive treatment guidance generally relies on the tuberculin test (TST) performance. However, due to its cross-reaction with non-tuberculous mycobacteria and the bacilli Calmette-Guérin (BCG) vaccine, its applicability is limited^3^. To avoid cross-reaction issues, interferon (IFN)-γ release assays (IGRAs) are the most recent and commonly used alternative for TB infection detection. IGRAs are based on the stimulation with specific *Mycobacterium tuberculosis* antigens followed by the measurement of the T-cell-mediated responses from peripheral blood lymphocytes (T-SPOT.TB; Oxford Immunotec Limited, Abingdon, UK) or whole blood (QuantiFERON technology; QFN, Qiagen, Düsseldorf, Germany)^4–6^. However, neither test type (TST and IGRAs) distinguish between active and LTBI, and therefore, do not provide evidence of recent infection, or risk of progression to active TB.

In order to improve LTBI diagnosis and not only distinguish between active disease and infection but also between the different infection stages, many studies have focused on the measurement of cytokines other than IFN-γ and also on the detection of a combination of cytokines^7–9^. In addition, the search of new *M. tuberculosis* antigens and characterization of the immune response after sample stimulation has also been extensively studied^10–15^. Some of the newly described antigens have been proven to yield different responses among active TB patients, TB exposed individuals and healthy controls^12,16,17^. However promising and innovative, there are contrary results among studies and more evidence is needed to validate the use of these novel *M. tuberculosis* antigens. Recently, Ruhwald et al. evaluated a promising ESAT-6 free IGRA which yielded a comparable performance to the QFN-G-IT^18^. In such study, several antigen and peptides were tested in order to find a combination that together with CFP-10, but lacking ESAT-6, could result in a comparable specific response to the currently used combination (ESAT-6, CFP-10 and TB7.7). After whole blood stimulation and specific response analysis, the most frequently recognized antigens by active TB and LTBI individuals were ESAT-6, CFP-10 and EspC followed by Rv2348-B and EspF. Recognition of these antigens was determined as the ability to produce more than 50 pg/ml of IFN-γ after antigen-specific stimulation. Those individuals that were able to recognize these antigens were considered as *responders.* As mentioned in this study, EspC, EspF and Rv2348-C were considered the most promising due to their magnitude of IFN-γ release and complementarity to CFP-10. Additionally, as described in this study, EspC, EspF and Rv2348 have less homology with non-tuberculous mycobacteria than ESAT-6 and CFP-10 and full sequence homology with the *M. tuberculosis* complex making them suitable for a TB infection diagnosis method with no cross-reaction issue^18^. Moreover, EspC recognized cases that were not detected by CFP-10 or ESAT-6 (11% and 12%, respectively).

Following the previous findings, the aim of this study was to test the effect of adding different combinations of EspC, EspF and Rv2348-B to the ESAT-6, CFP-10 and TB7.7 present in the QFN-G-IT assay, by measuring the amount of antigen-specific IFN-γ release in whole blood, in order to improve the accuracy of current TB infection diagnostic tests.

## Results

### Study population

A total of 115 subjects were included in this study from five different health care centres in Barcelona (Spain). A 64.3% (74/115) were active TB patients, 14.8% (17/115) were LTBI individuals and 20.9% (24/115) were healthy controls. For demographic and clinical data from the studied population see Table 1.

**Table 1:** Demographic and clinical data of the study population.

	Overall		
	(n = 115)		
	HC	LTBI	TB
	(n = 24)	(n = 17)	(n = 74)
**Age, average (years) ± SD**	37.0 ± 10.6	42.1 ± 9.9	41.1 ± 14.8
**Gender (%)**			
Female	20 (83.3)	4 (23.5)	21 (28.4)
Male	4 (16.7)	13 (76.5)	53 (71.6)
**Country of birth (%)**			
High TB burden^a^	0 (0.0)	2 (11.7)	11 (14.9)
Low TB burden	24 (100.0)	15 (88.2)	59 (79.7)
Unknown	0 (0.0)	0 (0.0)	3 (4.1)
**BCG vaccination (%)**	1 (4.2)	10 (58.8)	27 (36.5)
**QFN-G-IT**			
Positive	0 (0.0)	16 (94.1)	66 (89.2)
Negative	24 (100.0)	1 (5.9)^b^	8 (10.8)
**Prophylaxis (%)**			
Before starting prophylaxis	–	5 (29.4)	-
On prophylaxis (< 30 days)	0 (0.0)	12 (70.6)	0 (0.0)
Average (days) ± SD in patients with prophylaxis	–	21.1 ± 7.0	–
**Treatment (%)**			
Before starting treatment	–	–	9 (12.2)
On treatment	0 (0.0)	0 (0.0)	65 (87.8)
< 30 days	–	–	47 (63.5)
≥ 30 days	–	–	18 (24.3)
Average (days) ± SD in patients with treatment	–	–	24.09 ± 14.9

^a^About 150 cases per 100 000 population.

^b^T-SPOT.TB positive. HC: healthy controls, LTBI: latently tuberculosis infected individuals, TB: active tuberculosis patients.

### QFN-G-IT and in-house QFN

**Figure 1:**
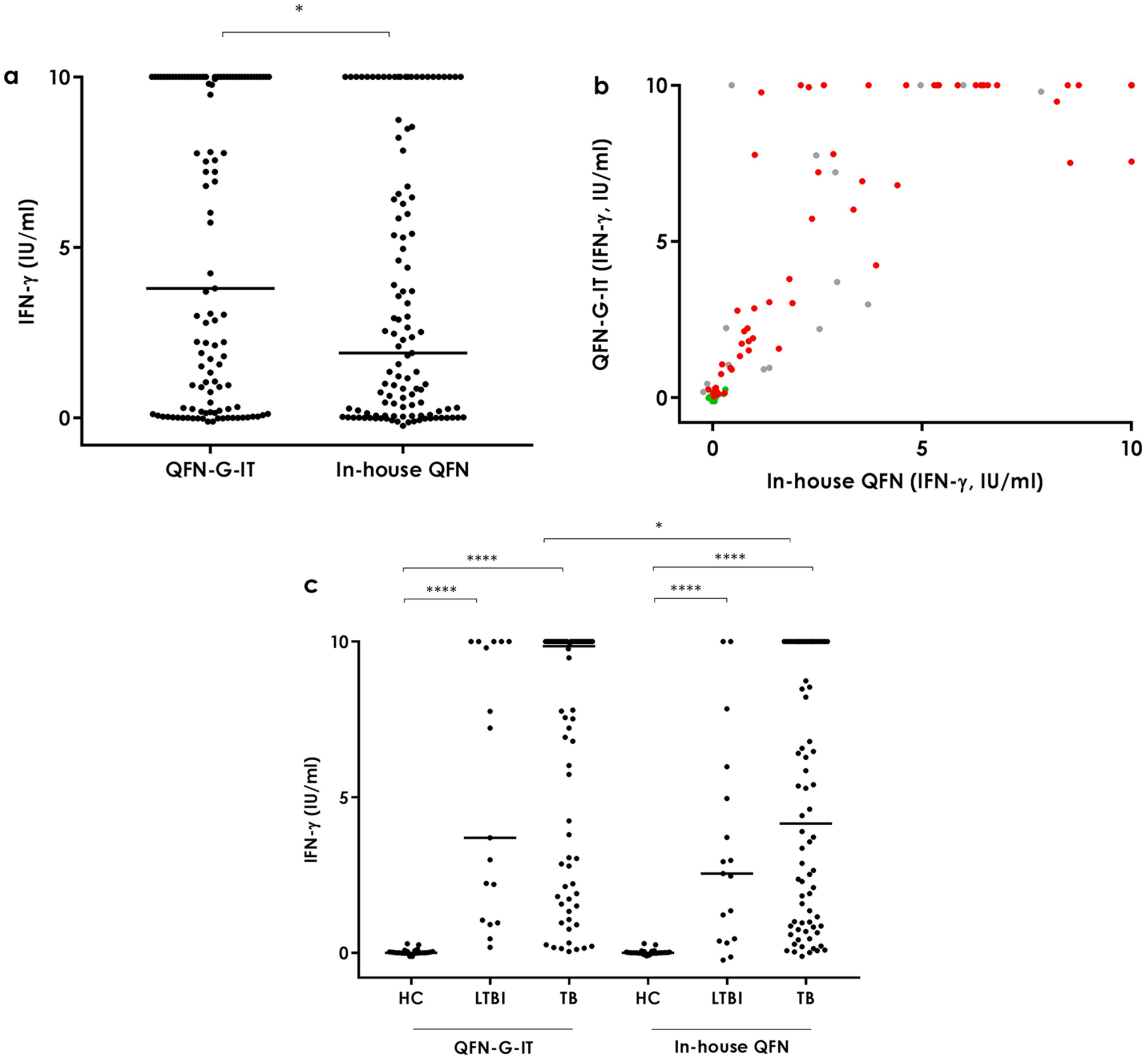
Antigen-specific IFN-γ release in the QFN-G-IT and in-house QFN tubes. **a** Overall. Median and interquartile range (IQR) values: 3.8 IU/ml (0.21–10.00) for the QFN-G-IT and 1.9 IU/ml (0.07–7.84) for the in-house QFN. **b** Overall correlation. Spearman r = 0.92. Green circles correspond to healthy controls, grey to LTBI individuals and red to active tuberculosis patients. **c** Per groups. Median and interquartile range (IQR) values: 0.005 IU/ml (-0.01–0.04), 3.7 IU/ml (1.005–10) and 9.855 IU/ml (2.073–10) for the QFN-G-IT in HC, LTBI and TB groups, respectively; 0 IU/ml (− 0.01 to 0.035), 2.55 IU/ml (0.415–5.47) and 4.155 IU/ml (0.86–10) for the in-house QFN in HC, LTBI and TB groups, respectively. *HC* healthy controls, *LTBI* latent tuberculosis infection, *TB* active tuberculosis. Differences between groups were analysed using Mann–Whitney U test (*p < 0.05, ****p < 0.0001).

Considering the overall study population, antigen-specific IFN-γ levels detected in the QFN-G-IT and in-house QFN tubes (both containing the same antigens: ESAT-6, CFP-10 and TB7.7) was significantly higher in the Qiagen tube than in the in-house (p < 0.05) due to differences in the active TB group (Fig. 1A,C). Nevertheless, the correlation between both tubes was very good (Spearman r = 0.92) (Fig. 1B). In both tubes, healthy controls had a significantly lower amount of antigen-specific IFN-γ production compared to active TB and LTBI (p < 0.0001) but no statistically significant differences were detected between LTBI and active TB samples (p > 0.05) (Fig. 1C).

### In-house QFN + A (+EspC, EspF and Rv2348-B) and in-house QFN

**Figure 2:**
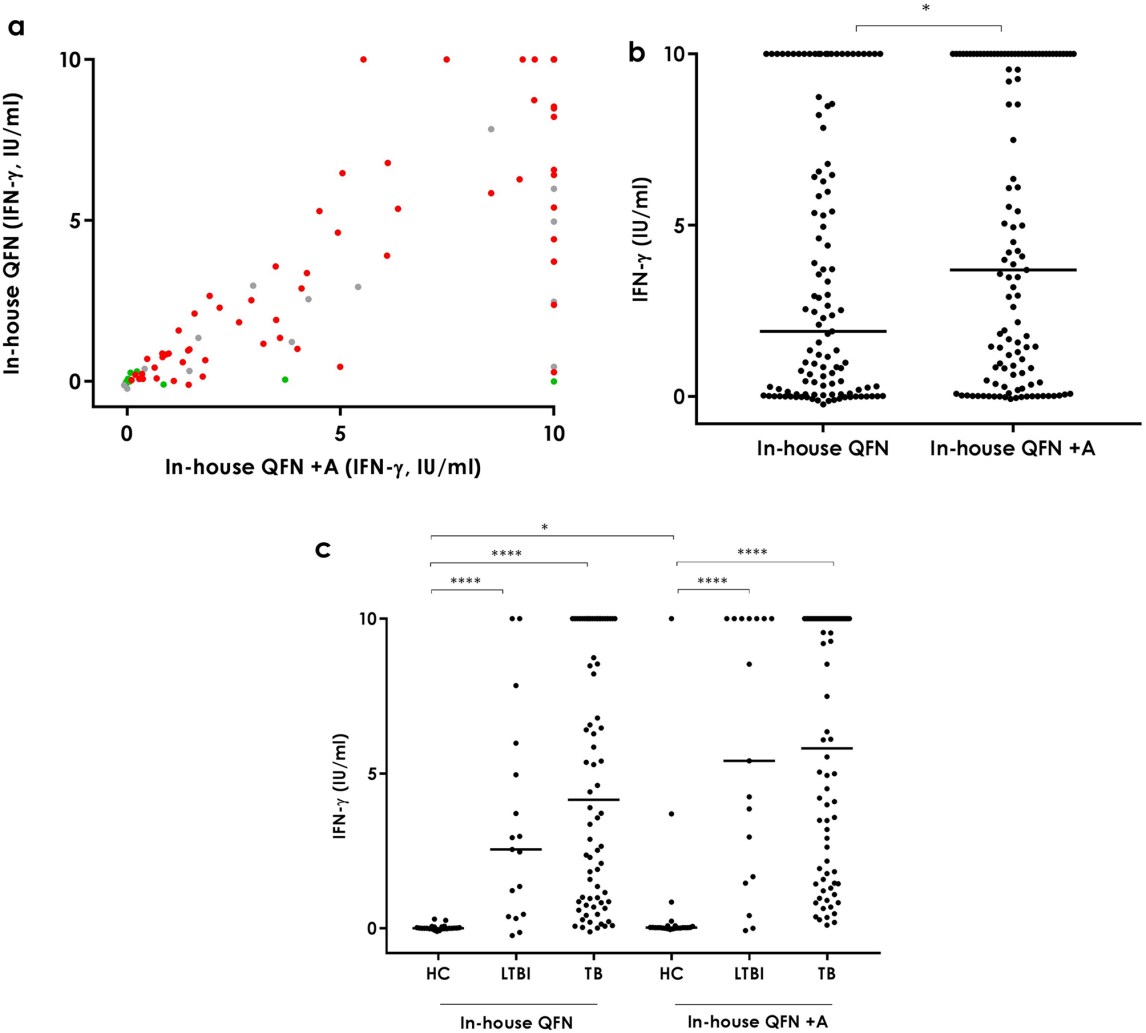
Antigen-specific IFN-γ release in the in-house QFN and in-house QFN + A tubes. **a** Overall correlation. Spearman r = 0.86. Green circles correspond to healthy controls, grey to LTBI individuals and red to active tuberculosis patients. **b** Overall. Median and interquartile range (IQR) values: 1.9 IU/ml (0.07–7.84) for the in-house QFN and 3.7 IU/ml (0.41–10.00) for the in-house + A. **c** Per groups. Median and interquartile range (IQR) values: 0 IU/ml (-0.01–0.035), 2.55 IU/ml (0.415–5.47) and 4.155 IU/ml (0.86–10) for the in-house QFN in HC, LTBI and TB groups, respectively; and 0.02 IU/ml (0.01–0.075), 5.41 IU/ml (1.565–10) and 5.815 IU/ml (1.55–10) for the in-house QFN + A in HC, LTBI and TB groups, respectively HC: healthy controls, LTBI: latent tuberculosis infection, TB: active tuberculosis. Differences between groups were analysed using Mann–Whitney U test (*p < 0.05, ****p < 0.0001).

In order to evaluate the benefits of adding three extra antigens (EspC, EspF and Rv2348-B) to the currently used stimulation (ESAT-6, CFP-10 and TB7.7) we compared the in-house QFN + A stimulation with the in-house QFN. Performance of the in-house QFN + A was similar to that of the in-house QFN with a strong overall correlation (Spearman r = 0.86; Fig. 2A) however, antigen-specific IFN-γ release was significantly higher (p < 0.05) in the in-house QFN + A than in the in-house QFN (Fig. 2B). If we exclude the three healthy controls that have a high response to the QFN + A antigen combination (Fig. 2C), this difference between the two tubes is no longer significant (data not shown). When classifying the study population in groups, healthy controls had a higher level of IFN-γ in the in-house QFN + A tube than in the in-house QFN (p < 0.05) (Fig. 2C). This statistically significant difference is again due to the above mentioned three healthy controls, pointing out at a possible specificity issue when using this antigen combination. No significant differences were detected among the active TB and LTBI groups. Moreover, as in the in-house QFN condition, IFN-γ levels in the in-house + A tube were higher in active TB patients (median [IQR] = 5.82 IU/ml [1.55–10.00]) and LTBI individuals (median [IQR] = 5.41 IU/ml [1.57–10.00]) than in healthy controls (median [IQR] = 0.02 IU/ml [0.01–0.08]) (p < 0.0001) (Fig. 2C).

### In-house QFN + B (+EspC and EspF) and + C (+EspC and Rv2348-B)

As described above, the addition of the three extra antigens present in the in-house QFN + A increased the number of positive results. In order to evaluate if the addition of not all three of the antigens but only two would also yield a similar response, we compared the in-house QFN + B and + C tubes with the in-house QFN + A.

**Figure 3:**
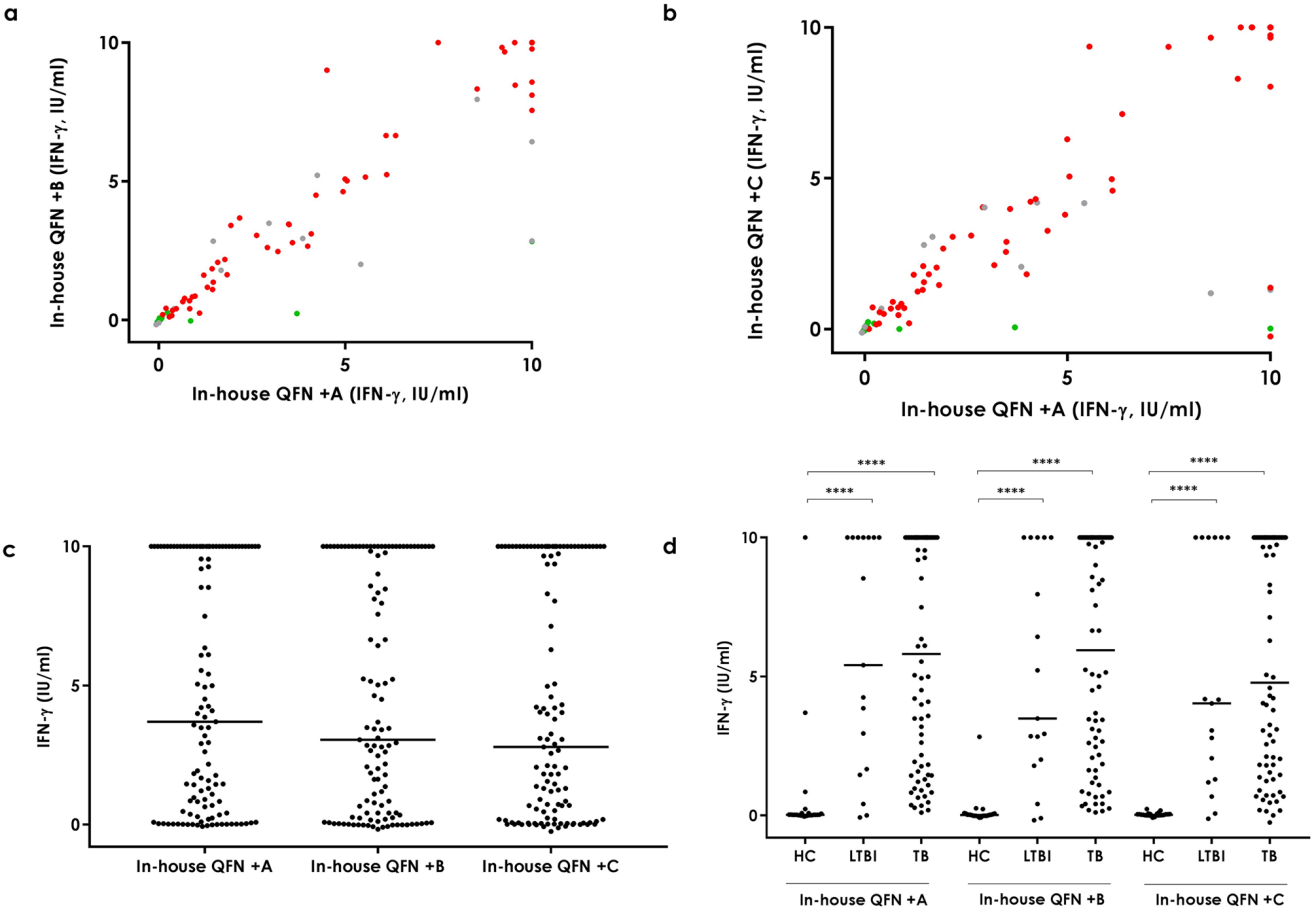
Antigen-specific IFN-γ release in the in-house QFN + A and in-house QFN + B and in-house QFN + C tubes. **a** Correlation between in-house QFN + A and + B. Spearman r = 0.95. Green circles correspond to healthy controls, grey to LTBI individuals and red to active tuberculosis patients. **b** Correlation between in-house QFN + A and + C. Spearman r = 0.87. Green circles correspond to healthy controls, grey to LTBI individuals and red to active tuberculosis patients. **c** Overall. Median and interquartile range (IQR) values: 3.7 IU/ml (0.41–10.00) for the in-house QFN + A, 3.05 IU/ml (0.26–10.00) for the in-house QFN + B, and 2.79 IU/ml (0.18–10.00) for the in-house QFN + C tube. **d** Per groups. Median and interquartile range (IQR) values: 0.02 IU/ml (0.01–0.075), 5.41 IU/ml (1.565–10) and 5.815 IU/ml (1.55–10) for the in-house QFN + A in HC, LTBI and TB groups, respectively; 0.015 IU/ml (-0.01–0.065), 1.9 IU/ml (1.9–10) and 5.945 IU/ml (1.795–10) for the in-house QFN + B in HC, LTBI and TB groups, respectively; and 0.025 IU/ml (0–0.05), 4.03 IU/ml (1.245–10) and 1.528 IU/ml (4.78–10) for the in-house QFN + C in HC, LTBI and TB groups, respectively. *HC* healthy controls, *LTBI* latent tuberculosis infection, *TB* active tuberculosis. Differences between groups were analysed using Mann–Whitney U test (****p < 0.0001).

Antigen-specific IFN-γ release in both extra in-house combinations correlated strongly with the in-house QFN + A (Spearman r = 0.95 when comparing in-house QFN + B with + A, and Spearman r = 0.87 when comparing in-house QFN + C with + A) (Fig. 3A,B). No significant differences were detected in the overall amount of antigen-specific IFN-γ release among tubes (Fig. 3C). As expected, IFN-γ levels were significantly higher in active TB patients (median [IQR]_in-house QFN +B_ = 5.95 IU/ml [1.80–10.00], median [IQR]_in-house QFN +C_ = 4.78 IU/ml [1.53–10.00]) and LTBI individuals (median [IQR]_in-house QFN +B_ = 3.49 IU/ml [1.90–10.00], median [IQR]_in-house QFN +C_ = 4.03 IU/ml [1.25–10.00]) than in healthy controls (median [IQR]_in-house QFN +B_ = 0.02 IU/ml [-0.01–0.07], median [IQR]_in-house QFN +C_ = 0.03 IU/ml [0.00–0.05]) (p < 0.0001) (Fig. 3D).

### ROC curves and positivity rates

**Figure 4:**
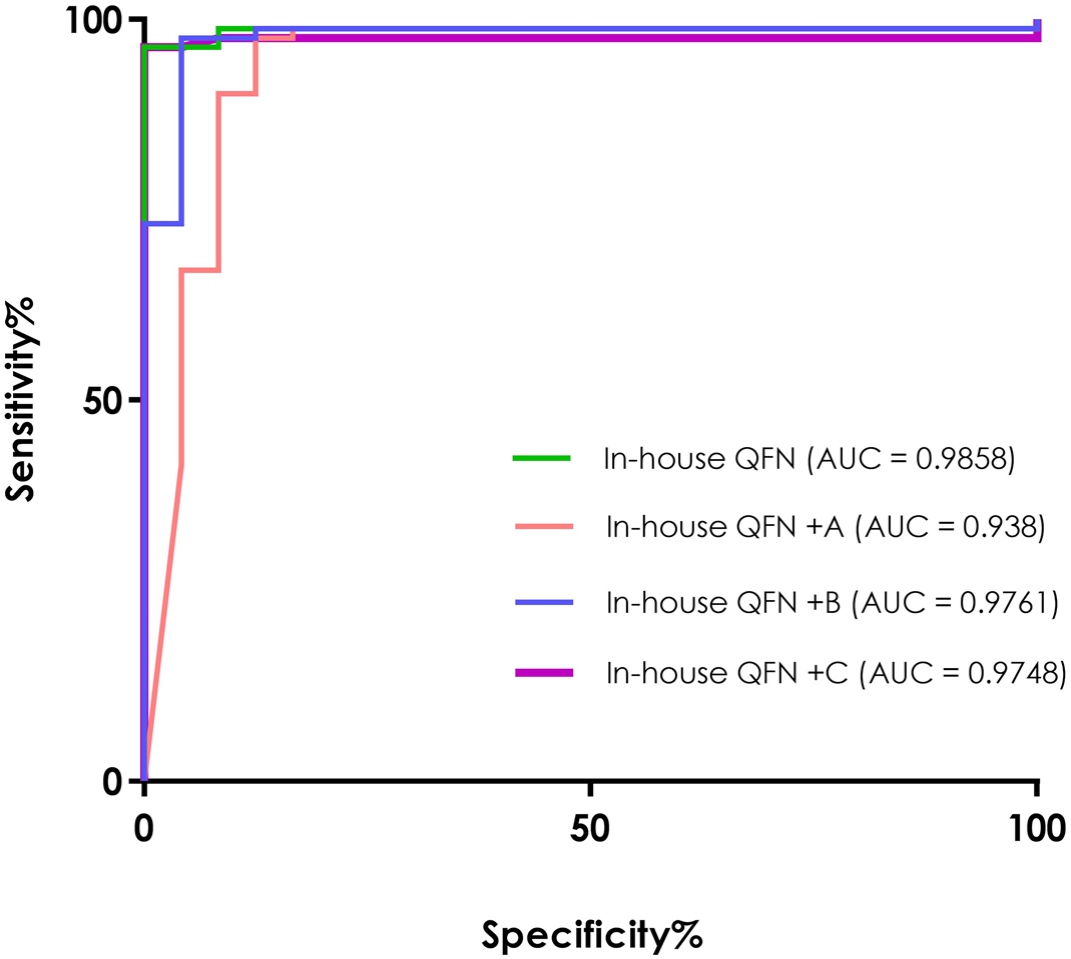
Receiver operating characteristic (ROC) curve analysis. Antigen-specific (antigen-nil) release of IFN-γ in the tested in-house tubes. Areas under the curve (AUCs) from each tested tube were comparable: in-house QFN AUC = 0.9858 (green line), in-house QFN + A AUC = 0.938 (orange line), in-house QFN + B AUC = 0.9761 (blue line) and in-house QFN + C AUC = 0.9748 (purple line). Active tuberculosis patients and latently infected individuals with a QFN-G-IT positive result were considered as positive controls and QFN-G-IT negative healthy controls as negative controls.

ROC curve analyses were performed in order to calculate the positivity cut-off values of the in-house QFN and in-house + A, + B and + C tubes. Those active TB and LTBI patients with a positive QFN-G-IT result were considered as positive cases and healthy controls (all of which had a negative QFN-G-IT) were considered as negative ones. The areas under the curve (AUC) were excellent, reaching 0.9858 in the case of the in-house QFN, 0.938 in the case of the in-house QFN + A, 0.9761 in the case of the in-house + B and 0.9748 in the case of the in-house QFN + C (Fig. 4).

Following the Youden’s Index (J), the suitable cut-off value for the in-house QFN condition was 0.31 IU/ml (J = 0.96) yielding a 96.34% sensitivity [CI: 89.68%—99.24%] and 100% specificity [CI: 85.75%—100%]. In the case of the in-house QFN + A tube, the selected cut-off was 0.29 IU/ml (J = 0.85) with a 97.56% sensitivity [CI: 91.47%—99.7%] and 87.5% specificity [CI: 67.64%—97.34%]. The selected cut-off for the in-house QFN + B was 0.335 IU/ml (J = 0.93) yielding a 97.56% sensitivity [CI: 91.47%—99.7%] and 95.83% specificity [CI: 78.88%—99.89%]. For the in-house QFN + C the cut-off was 0.345 IU/ml (J = 0.96) reaching a 96.34% sensitivity [CI: 89.68%—99.24%] and 100% specificity [CI: 85.75%—100%]. Interestingly, using the calculated positivity cut-offs, the overall amount of positive results obtained using the in-house QFN + A tube was higher than that obtained by the rest of the tested combinations QFN tubes (Table 2). The number of positive results obtained by the in-house QFN + B and in-house QFN + C was similar to that of the in-house QFN + A, being lower when using the in-house QFN + C (Table 2). Considering active TB and LTBI groups together, there was an overall of 7 more positive results using the in-house QFN + A (86/91) than using the in-house QFN (79/91), being all the 7 extra positives from the active TB group. However, there are also 3 healthy controls that yielded positive results in the in-house QFN + A that were QFN-G-IT and in-house QFN negative. Compared to the in-house QFN + B and + C, the in-house QFN + A combination yielded one positive result more (from the active TB group) than the in-house QFN + B (85/91) and two more than the in-house QFN + C (84/91).

**Table 2:** Test results obtained in the in-house QFN and in-house QFN + A, + B and + C tubes.

	Active TB (n = 74)	LTBI (n = 17)	Healthy Controls (n = 24)
**QFN-G-IT**			
Positive	66 (89.2)	16 (94.1)	0 (0.0)
Negative	8 (10.8)	1 (5.9)	24 (100.0)
**In-house QFN**			
Positive	64 (86.5)	15 (88.2)	0 (0.0)
Negative	10 (13.5)	2* (11.8)	24 (100.0)
**In-house QFN + A**			
Positive	71 (95.9)	15 (88.2)	3 (12.5)
Negative	3 (4.1)	2* (11.8)	21 (87.5)
**In-house QFN + B**			
Positive	70 (94.6)	15 (88.2)	1 (4.2)
Negative	4 (5.4)	2* (11.8)	23 (95.8)
**In house QFN + C**			
Positive	69 (93.2)	15 (88.2)	0 (0.0)
Negative	5 (6.8)	2* (11.8)	24 (100.0)

Cut-offs used: 0.35 IU/ml for the QFN-G-IT, 0.31 IU/ml for the in-house QFN, 0.29 IU/ml for the in-house QFN + A, 0.335 for the + B and 0.345 for the + C.

*One of them had a limit QFN-G-IT positive result and the other was also negative for QFN-G-IT. TB: tuberculosis, LTBI: latently tuberculosis infected individuals.

The agreement between tubes was considered as very good (κ = 0.938; CI = 0.869—1.000) when comparing the commercial and the in-house QFN tube and as good when comparing the QFN-G-IT with the in-house QFN + A (κ = 0.750; CI = 0.613—0.888) and both in-house tubes (κ = 0.781; CI = 0.655—0.908). There was a very good agreement between the in-house QFN + A and the + B (κ = 0.881; CI = 0.779- 0.983) and + C (κ = 0.837; CI = 0.721—0.953). Agreement between the in-house QFN + B and + C tubes was also very good (κ = 0.955; CI = 0.893—1.000).

## Discussion

LTBI diagnosis is key for disease control and program management; however, the current methods remain insufficient. Therefore, many efforts are invested in the development of new diagnostic tools as well as the improvement of the current ones. Following this direction, in this study, we evaluated the effect of adding extra specific antigens (EspC, EspF and Rv2348-B) to the ones currently used in the QFN-G-IT in order to test if their immunogenicity improved the assay’s performance increasing, therefore, the accuracy of the test.

Novel *M. tuberculosis* antigens have been extensively studied with the purpose to characterize better TB infection^10,12–14^. In this study, the immune response against new combinations of antigens was evaluated by measuring the levels of IFN-γ produced after specific stimulation. The antigenic combinations here tested rendered an IFN-γ response comparable to that obtained by the QFN-G-IT (Spearman r above 0.86). Stimulation with all the tested conditions yielded significantly higher IFN-γ levels in active TB patients and LTBI individuals compared to healthy controls (p < 0.0001). After ROC curve analysis and cut-off values selection, test results were calculated. The addition of EspC, EspF and Rv2348-B to the currently used ESAT-6, CFP-10 and TB7.7 enabled the identification of seven more positive cases. Addition of EspC and EspF identified less positive cases than when the three extra antigens were present but yielded more positive results compared to adding EspC and Rv2348-B (two more when considering the overall population and one when excluding healthy controls). These results show that the addition of EspC could increase the identification of positive cases together with EspF and Rv2348-B, being the latter antigen adding the least. However, the addition of these extra antigens could also increase the number of unspecific responses as seen in the in-house QFN + A and + B which detect as positive cases three and one healthy controls, respectively.

Recently, Whitworth et al. evaluated a second-generation ELISPOT based IGRA in which they added EspC to ESAT-6 and CFP-10^19^. In this study, they also come to the conclusion that by adding this extra antigen the diagnostic sensitivity increases compared to T-SPOT.TB and QFN-G-IT, with a mild decrease in specificity to 80%, similar to what we have obtained in this study (87.5% as the lowest specificity value). Although it is clear that the increase in specificity and sensitivity of the new antigen combinations are mild, these improvements could mean a big advance when considering HIV infected population^20^, patients under immunosuppressive therapy^21^, individuals with diabetes^22^, and children under 5 years of age^23,24^, which are population at high risk of developing TB and in which IGRAs performance can be affected by an impaired or immature immune response. In addition, the recent new version of the QFN-G-IT, the QuantiFERON-TB Gold Plus (QFN-Plus), has been described, so far, as equivalent to the QFN-G-IT in numerous studies done in various sites and considering different study populations ^25–28^. Considering that the addition of the antigens presented in this study does increase further the sensitivity of the QFN-G-IT test, and in the case of the in-house QFN + C with no specificity loss, their use to improve the current diagnostic tools should not be overlooked, especially when considering certain patient groups such as the above mentioned.

Given the immunogenicity of ESAT-6, this antigen has been extensively studied as a candidate component for protective and therapeutic vaccines^29–33^. Moreover, recently the use of this *M. tuberculosis*-specific antigen has been suggested and is already on trial as the next generation skin test together with CFP-10 in several studies^34–37^. The ESAT-6 and/or CFP-10 skin tests developed was shown to be safe and lack cross-reaction with the BCG vaccine and non-tuberculous mycobacteria.

In this context, the development of an ESAT-6 free IGRA that could serve as a companion diagnostic test in case of a future ESAT-6 based vaccine has been under study^18,38^. So far, the ESAT-6 free IGRA containing CFP-10, TB7.7, EspC, EspF and Rv2348-B, has yielded promising results, showing comparable performance to the QFN-G-IT. Although done in a small cohort of patients, it is worth mentioning that during this study we also tested this same ESAT-6 free combination in 9 samples (5 from active TB patients, 1 from LTBI individuals and 3 from healthy controls). Considering as responders those individuals with antigen-specific IFN-γ levels above 0.61 IU/ml (recently established cut-off)^38^, all healthy controls tested were negative, the LTBI individual was positive, and all active TB patients except one (with an IFN-γ release of 0.56 IU/ml) were positive. These results are similar to those described by Nemes et al.^38^ and Ruhwald et al^18^ with comparable specificity and sensitivity rates. These results, although in a very low number, add evidence to the previous studies done in this direction^18,19,38^ and emphasize the promising perspective of using this new antigenic combination for future TB diagnosis.

In this study, the immune response generated after stimulation with the different antigen combinations was evaluated solely by the amount of IFN-γ released. However, to characterize better this response, it would be of great interest to study the presence of other cytokines such as interferon gamma-induced protein 10 (IP-10), a combination of cytokines, and markers such as CD27, CD38, HLA-DR and Ki67^7,39–41^.

Furthermore, TST and IGRAs have been shown to have reduced sensitivity in patients at high risk of developing TB such as children, HIV positive individuals and patients with immune-mediated inflammatory diseases (IMIDs)^42,43^. Testing the antigen combinations used in this study with a cohort of high TB risk patients would be of great interest in order to characterize their immune response and compare it with that of the actual IGRAs, in search of an improved tool for these cohorts at risk.

The main limitation of this study is the fact that the evaluation of the performance of the antigen combinations tested has been compared to that of the QFN-G-IT which is not a gold standard test. Therefore, the cut-off value calculations and hence the specificity and sensitivity rates are affected by the result given using the QFN-G-IT.

Much is still to be done regarding TB control and management to decrease the disease’s burden. TB infection diagnosis, as well as stage characterization, are key for these tasks; however, the existing methods are not enough. In this study, we provided evidence that addition of EspC, EspF and (to less extent) Rv2384-B, to the current combination of ESAT-6, CFP-10 and TB7.7, could increase the detection of TB infected cases. Moreover, and based not only on this study but previous ones, we consider that the search for alternative stimulation combination for new TB diagnostic tests should focus on the use of an ESAT-6 free condition combined with the other specific antigens tested here. Evaluating different combination of specific immunogenic antigens such as EspC, EspF and Rv2348-B but lacking ESAT-6 and possibly CFP-10, is of great interest regarding the importance of the later vaccine. Moreover, evaluation of the immune response to such antigens by detection of cytokines other than IFN-γ alone should be done to evaluate their capacity not only to distinguish between TB infected and uninfected individuals, but also serve to distinguish between infection stages and disease and aid the diagnosis in high TB risk groups.

## Materials and methods

### Study population and ethical statement

In this study, we included whole blood samples from (i) microbiologically confirmed TB patients by means of a positive culture and/or a positive PCR, (ii) infected individuals (LTBI) (asymptomatic and with a positive IGRA) and (iii) healthy controls (uninfected individuals with a negative IGRA and no known contact with TB patients).

Such samples were collected at five medical centres in Barcelona, Spain: Unitat de Tuberculosi Vall d’Hebron-Drassanes, Unidad Clínica de Tratamiento Directamente Observado “Serveis Clinics”, Hospital Universitari Vall d’Hebron, Hospital Sant Joan Despí Moises Broggi, and Hospital Universitari Germans Trias i Pujol.

The study was approved by the ethical review board of the Ethics Committee of the Hospital Germans Trias i Pujol and subsequently for all the Ethics Committee of every participating health care centre (reference number CEI_PI-15-073) and by the ethical review board of the Capital Region of Copenhagen (reference number H-3-2012-008). Written informed consent was obtained from all study participants. Sample collection and all experiments were performed in accordance with relevant guidelines and regulations. Spare plasma samples were stored in the centre's approved biobank.

### Preparation of in-house tubes

Vacutainer blood extraction tubes were prepared by the Statens Serum Institut (SSI, Copenhagen, Denmark) with different antigen cocktails for blood stimulation. Briefly, 25ul containing 10 µg of the corresponding peptide mix (in-house QFN, QFN + A, QFN + B and QFN + C) together with 20 IU of heparin were added in cleaned 4 ml vacutainer tubes under sterile conditions. For the nil tube, 20 IU of heparin alone was added. Tubes were kept at -80ºC, then freeze-dried, recapped and vacuum for a total volume of 1 ml whole blood draw was done. The antigens used were selected based on their high rate of responders (antigen-specific release > 50 pg/ml IFN-γ)^18^. Briefly, EspC or Rv3615c, has been described as a target of cellular immunity in TB given its similar size and sequence homology to CFP-10 and ESAT-6^44^. EspF, or Rv3865 has also been described as a target for immunity in TB^45^ and been shown to induce substantial secretion of cytokines such as IFN-γ, IP-10, and TNF-α^13^. Finally, the RD7 encoded Rv2348, has been described as a potential contributor to pathogenesis^46^. More information about the selected antigens and which antigen is added in each in-house tube are shown in Table 3.

**Table 3:** Antigens used and in-house combinations.

	Rv number	Function	Protein length	Fraction assessed	Nil	QFN	QFN + A	QFN + B	QFN + C
ESAT-6	Rv3875	RD1, ESX1 substrate	95	1–95	–	Yes	Yes	Yes	Yes
CFP-10	Rv3874	RD1, ESX1 substrate	100	1–100	–	Yes	Yes	Yes	Yes
TB7.7	Rv2654c,p4	RD11, unknown	81	10–81	–	Yes	Yes	Yes	Yes
EspC	Rv3615c	ESX1 substrate	103	54–103	–	–	Yes	Yes	Yes
EspF	Rv3865	ESX1 associated protein	103	9-44	–	–	Yes	Yes	–
Rv2348-B	Rv2348-B	RD7, unknown	108	55-108	–	–	Yes	–	Yes

The previous antigens have the same length as described in Ruhwald et al. 2017^18^.

### Whole blood stimulation

Whole blood was drawn into the QuantiFERON-TB Gold In-Tube (QFN-G-IT; Qiagen, Düsseldorf, Germany) and the in-house tubes following the QFN-G-IT manufacturer’s instructions.

Briefly, 1 ml per tube was extracted from each patient. Tubes were placed in a roller for proper mixing of the antigens with the blood and afterwards, incubated overnight at 37ºC. After incubation, plasma was harvested and kept at -20ºC until IFN-γ determination.

### Quantification of IFN-γ production

The IFN-γ production was measured using the QFN-G-IT ELISA kit following the manufacturer’s instructions. Reactivity from both nil tubes (QFN-G-IT nil and in-house nil) was subtracted from the IFN-γ value in the corresponding QFN-G-IT and in-house tubes. In the case of the QFN-G-IT, a result was considered positive when the amount of IFN-γ was at least 0.35 IU/ml after antigen stimulation. A result was considered indeterminate when the stimulation was negative and the value of the positive control was less than 0.5 IU/ml or if the negative control was higher than 8.0 IU/ml. Mitogen from QFN-G-IT was used as the positive control of the in-house tubes. In the case of the in-house tubes, receiver operating characteristic (ROC) curves were performed in order to obtain cut-off values for each one.

### Statistics

Correlation among tested antigenic combinations was calculated using Spearman’s Rho correlation coefficient (r). Median and interquartile ranges (IQRs) were calculated for the levels of IFN-γ detected in each condition and differences in responses were compared using Mann–Whitney t-test, two-tailed. Differences among tests were considered statistically significant when p values were below 0.05. The diagnostic accuracy and cut-off values of the in-house tubes were determined using ROC curves and Youden’s Index (J). Calculation of agreement among tests and the 95% confidence interval (CI) was done using Cohen’s kappa (κ) coefficient. The strength of agreement was considered “perfect” when κ = 1.00, “very good” when κ values were between 0.99 and 0.81, “good” when κ values were between 0.80 and 0.61, and “moderate” when κ values were between 0.60 and 0.41, and “poor” when κ values were below 0.40. The software used for the statistical analyses and graphs were GraphPad Prism 7 (GraphPad Software Inc., La Jolla, CA, USA).

## Data Availability

Due to participant privacy, the data collected for this study is available upon request to the corresponding author.
